# Editorial: Recent advances in vector-borne diseases and climate change

**DOI:** 10.3389/fmicb.2025.1746230

**Published:** 2025-12-09

**Authors:** Mubasher Hussain, Yepin Yu, Liande Wang, Jawwad A. Qureshi

**Affiliations:** 1 Zhaoqing City Pest Control Engineering and Technology Center, Zhaoqing, Guangdong, China; 2 Guangdong Key Laboratory of Animal Conservation and Resource Utilization, Institute of Zoology, Guangdong Academy of Science, Guangdong, China; 3 State Key Laboratory of Ecological Pest Control for Fujian and Taiwan Crops, Key Laboratory of Biopesticide and Biochemistry, MOE, College of Plant Protection, Fujian Agriculture and Forestry University, Fuzhou, China; 4 Entomology and Nematology Department, Institute of Food and Agricultural Sciences (UF/IFAS), Southwest Florida Research and Education Center (SWFREC), University of Florida, Immokalee, FL, United States

**Keywords:** climate change, vector-borne diseases (VBDs), vector-pathogen interactions, epidemiological surveillance, public health

## Introduction

Vector-borne diseases continue to pose significant threats to the public health globally, with climate change exacerbating their transmission dynamics and expanding their geographic range. This Research Topic has brought together cutting-edge research that advances our understanding of the complex interactions between vectors, pathogens, hosts, and the changing environment. The 6 papers included in this Research Topic provide valuable insights into diverse aspects of vector-borne diseases, ranging from molecular mechanisms of vector-pathogen interactions to epidemiological studies on climate impacts and public health interventions.

## Vector-pathogen interactions at the molecular level

Two studies in this Research Topic delve into the intricate molecular interactions between vectors and pathogens. Chen et al. investigate the role of apoptosis and autophagy pathways in the interaction between the Asian longhorned tick (*Haemaphysalis longicornis*) and *Babesia microti*, the causative agent of human babesiosis. Their transcriptomic analysis reveals that *B. microti* infection significantly upregulates genes associated with these cellular processes in tick midgut tissues. Functional validation using RNA interference demonstrates that silencing of caspase-7, caspase-9, and ATG5 genes effectively reduces parasite burden, highlighting the pro-parasitic roles of these pathways in facilitating infection. This study provides novel insights into the molecular mechanisms underlying tick-borne pathogen transmission and identifies potential targets for intervention strategies.

In another molecular study, Singh Rajkumar et al. examined the transcriptomic changes in tomato-potato psyllid (*Bactericera cockerelli*) organs (salivary glands and ovaries) in response to infection with the phloem-limited bacterium *Candidatus Liberibacter solanacearum* (*C*Lso), a major plant pathogen. Their findings reveal organ-specific gene expression patterns, with salivary glands showing enrichment in processes related to neuronal transmission, cell adhesion, and respiration, while ovaries exhibit changes in DNA replication, transcriptional regulation, and stress responses. These distinct transcriptional signatures likely contribute to the horizontal and vertical transmission of *C*Lso, providing a foundation for developing new pest management strategies.

## Climate change impacts on disease transmission

The influence of climate change on vector-borne disease dynamics is a central theme of this Research Topic. Haq et al. present a comprehensive analysis of malaria incidence in the Bannu district of Pakistan, demonstrating significant associations between climatic factors (temperature, rainfall, and humidity) and disease occurrence. Using Poisson regression models, they show that a 1 °C increase in temperature is associated with a 4% increase in malaria incidence, while a 1% increase in humidity increases incidence by 2%. Topographic variables also play a crucial role, with higher elevation areas showing increased transmission risk. These findings emphasize the importance of integrating climatic factors into malaria control strategies.

Zhang et al. provide a global perspective on how climate change affects mosquito-borne diseases such as malaria, dengue, Zika, chikungunya, and yellow fever. Their review highlights the multifaceted impacts of rising temperatures, altered rainfall patterns, and extreme climatic events on mosquito biology, pathogen development, and disease transmission. The authors note the expanding geographic range of vectors like *Aedes albopictus* into temperate regions of Europe and North America, as well as the increased incidence of dengue in Southeast Asia linked to changing monsoon patterns. The review also discusses innovative control strategies, including Wolbachia-infected mosquitoes, genetically modified mosquitoes, and sterile insect techniques, emphasizing the need for integrated approaches.

## Epidemiological surveillance and public health

Two studies focus on epidemiological surveillance and public health aspects of vector-borne diseases. Orf et al. utilize next-generation sequencing (NGS) to identify pathogens associated with non-malarial acute febrile illness (AFI) in Senegal. Their metagenomic approach detected a diverse array of pathogens in 22% of malaria-negative specimens, including *Borrelia crocidurae*, West Nile virus, *Rickettsia feli*s, and *Bartonella quintana*. Notably, they observed distinct seasonal patterns, with mosquito-borne pathogens detected after the rainy season and tick-borne pathogens found before the rainy season. This study demonstrates the utility of NGS for comprehensive pathogen surveillance and outbreak detection.

Siddique et al. investigate knowledge and perceptions of climate change and its link to dengue among Bangladeshi youth. Their cross-sectional study of 1,358 participants reveals moderate levels of climate change knowledge (mean score 7.10 ± 3.20, out of 13 items) but strong recognition of the dengue-climate connection (mean score 26.60 ± 4.12, out of 33 items). Factors associated with higher knowledge and positive perceptions include unmarried status, nuclear family structure, non-smoking, good self-perceived health, regular sleep patterns, moderate social media use, and daily media consumption. The study highlights the importance of targeted educational campaigns to enhance climate consciousness and dengue prevention among youth.

## Conclusion and future directions

The studies presented in this Research Topic collectively advance our understanding of vector-borne diseases in the context of climate change. They highlight the complexity of vector-pathogen interactions at molecular, ecological, and epidemiological levels, and underscore the urgent need for integrated approaches to disease control.

Future research should focus on: (1) developing predictive models that incorporate climate data to forecast disease outbreaks; (2) identifying novel molecular targets for transmission-blocking interventions; (3) enhancing surveillance systems using advanced technologies like NGS; and (4) implementing community-based educational programs to improve public awareness and engagement.

As climate change continues to reshape the landscape of vector-borne diseases, interdisciplinary research and global collaboration will be essential to mitigate their impact on public health, agriculture, and ecosystems. This Research Topic provides a foundation for these efforts, bringing together insights that can inform evidence-based policies and interventions to address this growing global challenge.

